# Insect-derived polymer hydrogel based on fibroin matrix from whole silkworm larvae

**DOI:** 10.1371/journal.pone.0335864

**Published:** 2025-11-07

**Authors:** Maki Yamazaki, Aoi Tojo, Shusuke Hashimoto, Masakazu Kobayashi, Kenjiro Yazawa, Kunihiro Shiomi

**Affiliations:** Faculty of Textile Science and Technology, Shinshu University, Ueda, Japan; Baba Kinaram Research Foundation, INDIA

## Abstract

Insect-derived polymers, known for their mechanical properties, biocompatibility, and sustainability, are increasingly used in pharmaceuticals, food, and tissue engineering. *Bombyx mori*, the silkworm, produces silk fibroin (SF) and sericin, both crucial for biomaterial development. In this study, we aimed to prepare a hydrogel derived from whole silkworm larvae powder (B100rw) without additional gelling agents and to investigate its gelation behavior. The gelation process was analyzed by examining the compressive stress, adhesiveness, and gelation time under low-temperature conditions. Structural characterization was performed using Fourier-transform infrared spectroscopy and wide-angle X-ray scattering to determine the β-sheet content and crystallinity of the hydrogel. The gelation behavior and mechanical properties of B100rw and SF hydrogels were compared. The B100rw hydrogel exhibited gelation primarily due to fibroin heavy chain (FibH), as evidenced by the failure of gelation when *FibH* was knocked out in the larvae. The hydrogel demonstrated significantly higher compressive stress and adhesiveness at low temperatures than SF hydrogels, with faster initial gelation. Structural analysis revealed that the B100rw hydrogel possessed a β-sheet conformation similar to SF hydrogels but with lower crystallinity. The presence of additional insect-derived polymers like sericin, chitin, and cellulose in B100rw likely contributed to these enhanced gelation properties. This study successfully developed a novel hydrogel from B100rw, demonstrating distinct gelation behavior and unique mechanical properties compared to traditional SF-based hydrogels. The findings suggest that B100rw-derived hydrogels could be used as multifunctional platforms for food and medical applications, leveraging the natural gelling properties of insect-derived polymers. Further research into optimizing the gelation process and exploring alternative insect-derived polymer hydrogels could enhance the potential of these materials for biotechnological applications.

## Introduction

Insects and their derivatives are increasingly utilized across diverse fields, including pharmaceuticals, tissue engineering, dentistry, agriculture, veterinary medicine, cosmetics, cosmeceuticals, food, and nutraceuticals [1]. Recent advancements in biomaterials development incorporating insect-derived polymers have demonstrated exceptional mechanical properties, sustainability, and biocompatibility through innovative strategies. These biomaterials offer versatile platforms for medical applications and can also serve as hosts for various other uses [2,3].

*Bombyx mori* silkworm cocoons comprise 75–83.3% silk fibroin (SF) and 16.7–25% sericin. SF consists of three main proteins: fibroin heavy chain (FibH), fibroin light chain (FibL), and a glycoprotein, P25/fibrohexamerin, with molecular masses of 392, 26, and 30 kDa, respectively. These proteins form a complex known as the elementary unit, with a molar ratio of 6:6:1. In this structure, FibH and FibL form a heterodimer stabilized by a single disulfide bond at the C-terminal domain of FibH, while the interaction between FibH, FibL, and P25/fibrohexamerin is non-covalent [4]. Among these, FibH accounts for approximately 90% of the molecular weight of the fibroin complex; therefore, it is speculated that FibH plays a crucial role in determining the physical and chemical properties of SF. The FibH sequence consists of an N-terminus, a C-terminus, and a central region containing a highly repetitive GAGAGS segment (where G = glycine, A = alanine, and S = serine) (S1 Fig) [5]. Therefore, SF, predominantly containing FibH, is a distinctive hydrophobic protein rich in glycine and alanine.

SF has been explored as a potential biopolymer due to its favorable physicochemical properties, such as excellent biocompatibility, biodegradability, bioresorbability, low immunogenicity, and tunable mechanical properties [6]. SF-based biomaterials can be fabricated into films, hydrogels, sponges, 3D structures, and nanoparticles [6,7]. SF hydrogels, which consist of water-swollen 3D polymer networks, are formed via physical or chemical crosslinking, making them ideal for applications in tissue engineering, in vitro disease models, and drug delivery systems [8–11]. During the sol-gel transition, SF solutions undergo a conformational shift from a random coil to a β-sheet structure [6]. The gelation kinetics of SF hydrogels is influenced by several factors, such as temperature, sonication, pH, protein concentration, and the addition of precipitating agents, or by synergistically combining SF with other polymers [9]. Moreover, transgenic silkworm platforms have been used to generate genetically engineered SF hydrogels with tailored properties [8,12,13]. Consequently, silk-based biomaterials have evolved beyond traditional textile applications to advanced applications, such as hydrogel platforms, in high-tech sectors [3].

In our previous study, we evaluated the bio-physicochemical properties of raw powder prepared from dried whole silkworm larvae, designated as B100rw [14]. The yield of B100rw was nearly 100% and contained approximately 25% SF, along with other proteins derived from silkworm larvae and mulberry leaves. Additionally, B100rw maintained trehalase activity, serving as an indicator enzyme. The powder also retained a random coil conformation similar to that of liquid silk. These findings suggest that B100rw preserves the unique physicochemical properties of live larvae. Furthermore, an *ad libitum* diet containing B100rw altered the composition and diversity of the cecal and fecal microbiota in mice compared to the control [15].

Based on these findings, we speculated that B100rw contains naturally occurring polymers like fibroin, sericin, chitin, and cellulose. These natural polymers could be blended to form novel functionalized hydrogels without the need for additional gelling agents. For instance, sericin undergoes gelation by transitioning from a random coil to a β-sheet structure at low temperatures (10°C), forming a three-dimensional (3D) matrix. This process not only promotes sericin hydrogel formation but also enables reversibility to a sol phase when heated (50–60°C) [16,17]. Building on these findings, we hypothesized that B100rw may serve as a platform for multifunctional materials in medical and food applications.

To test this hypothesis, in this study, we aimed to prepare hydrogel using B100rw. Specifically, we examined the role of FibH in gelation and compared the properties of B100rw hydrogels with those of traditional SF-based hydrogels. These results offer insights into the gelation mechanisms of silkworm-based biopolymers, which may find applications in the biomedical and food industries.

## Materials and methods

### Silkworm, cocoon, and mulberry leaves

The Kosetsu strain of *B. mori* was used in this study. Wild-type (WT) and *FibH* knockout (KO; Acc. No. AF 226688) strains were reared on mulberry leaves and an artificial diet, respectively, and subsequently collected. Some larvae were allowed to spin cocoons, which were collected before adult eclosion. Mulberry leaves were collected from a mulberry garden at Shinshu University (https://www.ftst.jp/farm/). Collected larvae and mulberry leaves were immediately washed with tap water, frozen in liquid N_2_, and stored at −20°C until use.

### Clustered regularly interspaced short palindromic repeats- associated protein 9 (CRISPR-Cas9) and screening of KO silkworm

To generate *FibH* KO mutant, CRISPR RNA (crRNA) containing the target sequence 5′-CCTTTGTGATCTTGTGCTGCGCT-3′ (S1 Fig), was designed using CRISPRdirect software (https://crispr.dbcls.jp/). Alt-R CRISPR-Cas9 crRNA, Alt-R CRISPR-Cas9 trans-activating crRNA (tracrRNA), and Alt-R Cas9 enzymes were purchased from Integrated DNA Technologies (Coralville, IA, USA). The ribonucleoprotein complex was prepared according to the Alt-R CRISPR-Cas9 system and injected into newly laid eggs, as described in a previous study [18]. Briefly, non-diapause eggs were collected within 1 h of oviposition during the syncytial blastoderm stage, and a mixture of crRNA and Cas9 was injected into the eggs using a glass needle (uMPm-02; Daiwa Union, Iida, Japan) attached to a manipulator (kaikopuchu-STDU1; Daiwa Union) and FemtoJet (Eppendorf, Tokyo, Japan).

For germline mutagenesis screening, G_0_ adults were mated with WT adults. Oviposited G_1_ eggs were collected, and approximately 10 eggs per brood were pooled for genomic DNA extraction using DNAzol reagent (Thermo Fisher Scientific, Waltham, MA, United States). DNA fragments containing the targeted region of interest were amplified by PCR using PrimeSTAR Max DNA Polymerase (Takara, Tokyo, Japan) and specific primers (Forward; 5′-CATAATTAATCACATTGTTCATG-3′ and Reverse; 5′-CAGCACTAGTGCTGCAGTCGGTGC-3′). Mutations were detected using a T7 Endonuclease I reaction assay (Nippon Gene Co., Tokyo, Japan) and confirmed by sequencing. Broods carrying mutated sequences were reared, and mutated G_1_ adults were crossed with siblings carrying the same mutation to produce homozygous mutants, verified by sequencing the target regions in G_2_ or G_3_.

### Preparation of powder and hydrogel

**Fig 1:**
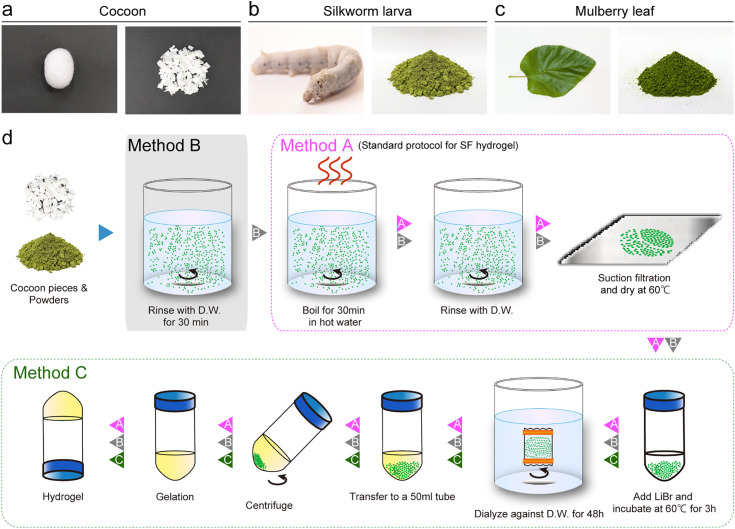
Schematic of the silkworm larva (B100rw) hydrogel preparation. **(a–c)** Preparation of (**a**) cocoon pieces and powders of (**b**) silkworm larva and (**c**) mulberry leaf for hydrogel preparation. (**d)** Schematic diagram showing the methods of hydrogel preparation (methods A, B, and **C)**. In method A (pink arrowheads), each powder was boiled for 30 min with hot water, followed by rinsing with distilled water (D.W.), suction filtration, and drying at 60°C. The extract was dissolved in 9.3 M lithium bromide (LiBr) solution at 60°C for 3 **h.** The solution was dialyzed for 2 days with **D.**W. using a dialyzed membrane. The soluble fraction of hydrogel was obtained by centrifugation. Method A was slightly modified in method B and C; in method B (black arrowheads), the powder was suspended in **D.**W. for 30 min with thorough mixing before boiling, whereas in method C (green arrowheads), the boiling and rinsing steps were omitted. This schematic diagram is adapted from a previous study [7] with some modifications.

B100rw and mulberry leaf powder were prepared according to the procedure described in a previous study [14]. Briefly, frozen silkworm and mulberry leaves were lyophilized using a freeze-dryer (FDM-2000; EYELA, Tokyo, Japan). Dried samples were pulverized using an MB3000 Multi-Beads Shocker (Yasui Kikai, Osaka, Japan) at 3,000 rpm for 20 s. The cocoons were cut into 5 mm square pieces using scissors (Fig 1a-c).

To prepare the hydrogel, we used three methods (A, B, and C) based on SF extraction from cocoons [7,19] (Fig 1d). In method A, each powder (7 g) and cocoon pieces (1 g) were used as starting materials for gelation. The samples were boiled in 1 L distilled water for 30 min, collected using a Buchner funnel, rinsed with 1 L distilled water, and thoroughly dried on aluminum foil. The dried extracts and cocoon pieces were dissolved in a 9.3M lithium bromide (LiBr) solution at 60°C for 3 h to produce a 10% (w/v) solution with an equal volume of silk gland. This solution was dialyzed for 2 days against distilled water (3 L) using a SnakeSkin dialysis membrane (Thermo Fisher Scientific) with a molecular weight cutoff of 3.5 kDa, with five water changes. The soluble fraction was then collected by centrifugation at 9000 rpm (12,700 × *g*) for 20 min at 4°C.

To prepare hydrogels with other properties, we examined additional protocols (methods B and C). In method B, to eliminate soluble proteins from the LiBr extract, B100rw was suspended in 1 L of distilled water for 30 min with thorough mixing before boiling. In method C, the boiling step was omitted to retain various components in LiBr extract (Fig 1d). The protein content was determined using the combustion method with DUMATHERM Npro (C. Gerhardt Japan, Tokyo, Japan) and a protein-to-nitrogen conversion factor of 6.25.

Each LiBr extract was placed in a 15 mL tube and sonicated at 37°C for 10 min using an ultrasonic cleaning machine (US-2KS; SND, Nagano, Japan). An aliquot (1 mL) of each extract was placed in a 4 mL flat bottom vial and incubated at 37°C for 3 days. Gelation was assessed by inverting the vial and observing whether the sample remained immobile [20]. The compressive stress of the hydrogels was measured at different time points as described in the Mechanical properties section.

### Mechanical properties

The mechanical properties of the hydrogels were evaluated using texture profile analysis, as described in previous studies [20,21]. Each LiBr extract (2 mL) was placed in a 24-well flat-bottom microplate (IWAKI; AGC Techno Glass Co., Ltd., Shizuoka, Japan) and incubated at 37°C for 1–4 days or at 4°C for 1–21 days. Each well had a cross-sectional diameter of 15 mm and a height of 10 mm. Texture profile analysis was performed at 20–25°C using a CREEP METER RHEONER II RE2-33005C (XZ) instrument (YAMADEN, Tokyo, Japan) equipped with a 2N or 200N load cell. The samples were subjected to two-cycle compression; in each compression cycle, the sample was compressed to 2/3 of its original height using an 8 mm diameter plunger operated at 10 mm/s. The maximum force during the first compression (compressive stress) and negative maximum force after the first compression (adhesiveness) were recorded. Furthermore, to assess the effect of ultrasonic treatment on gelation, the gel was sonicated for different durations (10; 30; 60; and 1,800 s) at 37°C, dispensed into a 24-well flat-bottom microplate, incubated at 37°C for 1 day, and then subjected to texture profile analysis.

### Sodium dodecyl sulfate-polyacrylamide gel electrophoresis (SDS-PAGE) and proteomic analysis

Each LiBr extract was mixed with sample buffer at a ratio of 1:1 (v/v) and electrophoresed on a 5–20% polyacrylamide gradient gel (EHR-T520L; ATTO Corp., Tokyo, Japan) at 300 V for 50 min. Precision Plus Protein Kaleidoscope Standards (Bio-Rad, Hercules, CA, USA) were used as the protein standards. The gels were stained with CBB R-250 (FUJIFILM Wako, Osaka, Japan), and protein bands or areas of interest on the SDS-PAGE gel were excised. The gel pieces were then treated with 10 mM dithiothreitol and alkylated with 55 mM iodoacetamide, followed by digestion with 12.5 mg/μL trypsin overnight at 37°C. The resulting peptides were separated using a 0–40% linear acetonitrile gradient for 30 min, followed by liquid chromatography-tandem mass spectrometry (LC-MS/MS) analysis (nanoACQUITY UPLC Xevo QTOF; Waters, MA, USA). Data were processed using ProteinLynx Global Server 3.0.3 and searched against *B. mori* and *Morus* spp. L. (mulberry) protein entries in the UniProt Knowledgebase (UniProtKB; https://www.uniprot.org/uniprotkb/).

### Attenuated total reflectance/Fourier transform infrared (FT-IR) spectral analysis

Protein secondary structures in the gelling samples were analyzed using an FT-IR device (IR Prestige-21; Shimadzu Corp. Kyoto, Japan) equipped with an attenuated total reflectance (ATR) accessory (DuraSamplIRⅡ, Smiths Detection, London, England). The spectra for each sample were recorded with 30 accumulations at a resolution of 4.0 cm^−1^ over a wavenumber range of 500–4000 cm^−1^ [19]. The collected spectra were corrected using water as background.

### Wide-angle X-ray scattering (WAXS)

Each LiBr extract was sonicated for 10 min at 37°C, and incubated at 37°Cfor 3 days.After gelation, the samples were frozen at −20°C, lyophilized using a freeze dryer (FDM-2000; EYELA, Tokyo, Japan), and further dried at −80°C under 4 Pa vapor pressure in a dry chamber (DRC-3 L; EYELA) for 24 h. WAXS of the lyophilized samples was measured using X-ray radiation with an energy of 12.4 keV and a wavelength of 0.1 nm at beamline BL-10C at the Photon Factory (Tsukuba, Japan; https://www2.kek.jp/imss/pf/eng/) [19]. Each scattering pattern was detected with an exposure time of 15 s, and the sample-to-detector distance was set to 238 mm. The resulting scattering data were radially integrated and converted into one-dimensional profiles using Fit2D software [22]. The obtained data were corrected by subtracting the background scattering. The crystallinity was evaluated based on the area of the crystal peaks divided by the total area of the amorphous halo and the crystal peaks by fitting with the Gaussian function using Igor Pro 8.03 (WaveMetrics, Inc., Portland, OR, USA).

### Statistical analysis

Statistical parameters, including definitions and *n* values, are provided with the relevant figures or corresponding figure legends. Statistical analyses were performed in Excel 2011 (Microsoft) using the Toukei–Kaiseki Ver. 3.0 add-in (Esumi). Data are expressed as mean ± standard deviation (SD). Differences with a *p*-value of < 0.05 were considered statistically significant.

## Results

### Gelation of silkworm larval powder (B100rw)

**Fig 2:**
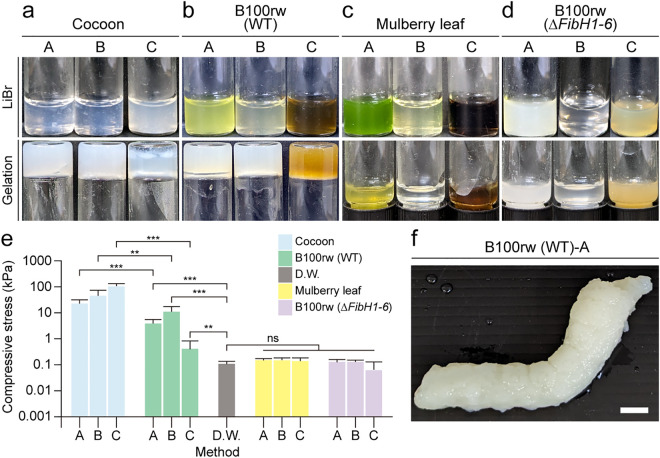
Gelation of cocoon pieces, B100rw powder, and mulberry leaf powder. LiBr extracts prepared from (**a**) cocoon, **(b)** B100rw (WT), (**c**) mulberry leaf, and **(d)** B100rw (∆*FibH1-6*) using methods A, B, and **C.** For gelation, each LiBr extract was sonicated at 37°C for 10 min, then 1 mL of each extract was placed in a 4 mL flat bottom vial and incubated at 37°C for 3 days (LiBr). The vial was inverted, and its immobility was observed to assess the gelation (Gelation). **(e)** Compressive stress of each hydrogel prepared using the three methods. Each LiBr extract was sonicated at 37°C for 10 min and then incubated at 37°C for 3 days to measure the compressive stress. **(f)** Silkworm larva-shaped hydrogel made from B100rw using method **A.** The LiBr extract of B100rw prepared using method A was sonicated for 10 min at 37°C, then poured into a silkworm larvae mold and incubated at 37°C for 3 days. Data represent the mean ± standard deviation (SD) of five samples. Statistical differences were determined using a Student’s *t*-test. ns, non-significant; ** *p* < 0.01; *** *p* < 0.001; Scale bar, 1 cm.

Evaluation of the ratio of the weight of the silk gland to that of the whole body of the Kosetsu strain on a dry weight basis revealed that the weight of the silk gland was approximately 17%, accounting for approximately 1/7 of the whole body weight (S1 Table). Therefore, 1 g of the cocoon layer and 7 g of B100rw were used as the starting materials for gelation so that the SF contents of the cocoons and B100rw were comparable. Furthermore, the weight of mulberry leaves was 7 g, which was equivalent to that of B100rw. The schematic of the preparation of hydrogels using cocoon pieces, B100rw, or mulberry leaf powder is presented in Fig 1. The cocoon-derived hydrogel (SF hydrogel) developed an opaque white color. It did not fall from the inverted tube (Fig 2a).

Similarly, gelation was observed in the hydrogel prepared using B100rw (WT) but not in that prepared using mulberry leaf powder (Fig 2b, c). Next, we examined whether the main gelling agents in the B100rw hydrogels were SF. Therefore, we constructed a *FibH* KO mutant, which consists of an SF complex (S1 Fig). The truncated mutant, ∆*FibH1-6,* comprised an insertion of 22 bp in the N-terminal region of *FibH* to ensure that it encodes the 10 amino acids. The hydrogels derived from B100rw (∆*FibH1-6*) did not show gelation in any of the three methods (Fig 2c). It is known that the repetitive and non-repetitive regions of FibH participate in the physical gelation ability of the SF hydrogel [9]. Therefore, we speculated that the gelation of B100rw hydrogels was mainly due to SF.

The compressive stress of B100rw (WT) hydrogel obtained using the three methods (B100rw-A, -B, and -C) was statistically lower than that of the SF hydrogel obtained using the same method (Fig 2e and S1 File), although the SF content was similar in all extracts. Furthermore, our findings revealed that B100rw (WT)-derived hydrogel could be molded into various shapes (Fig 2f).

### Gelling properties of B100rw in initial reaction

**Fig 3:**
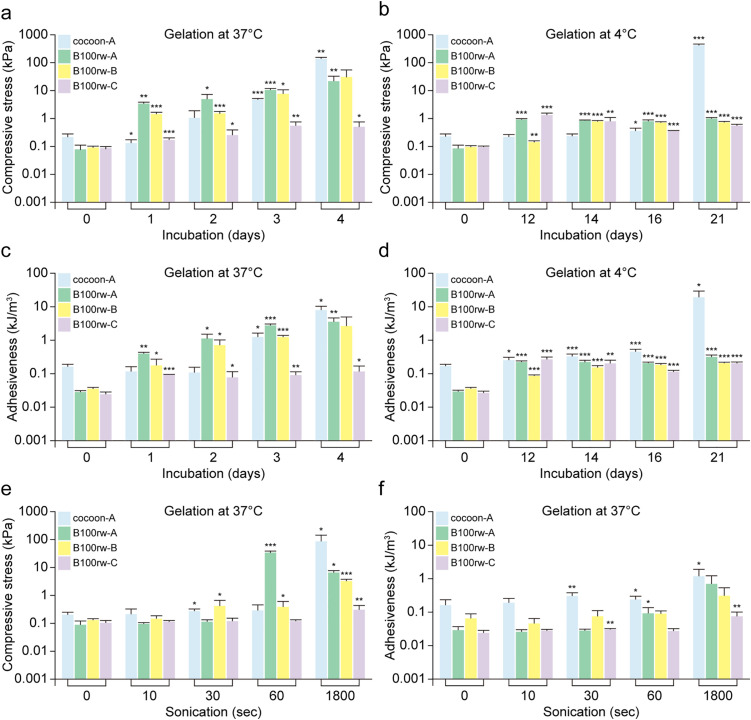
Mechanical properties of cocoon-A, B100rw-A, -B, and -C hydrogels. **(a–d)** Effect of the indicated incubation durations on (**a**, **b**) compressive stress and (**c**, **d**) adhesiveness of hydrogels incubated at 37°C (**a**, **c**) and 4°C **(b, d)**. **(e–f)** Effects of indicated sonication durations on (**e**) compressive stress and (**f**) adhesiveness of the hydrogels incubated at 37°C for 1 day. Data represent the mean ± standard deviation (SD) of five samples. Statistical differences were assessed using Student’s *t*-test. * *p* < 0.05; ** *p* < 0.01; *** *p* < 0.001.

Next, we investigated the effects of the gelling factors (temperature and sonication time) on hydrogel properties (compression stress and adhesiveness) during the initial gelation (Fig 3 and S2 File). The compression stress of SF hydrogel (cocoon-A) prepared from cocoon using method A incubated at 37°C increased significantly on day 3 compared to that of the gel on day 0 (Fig 3a). In the B100rw hydrogels, the compression stress significantly increased on day 1 after incubation, irrespective of the method of preparation, suggesting that the gelation speed was faster than that of the SF gel (Fig 3a). The compression stress of all the B100rw hydrogels showed a significant increase on day 12 of incubation (Fig 3b). These findings indicated different properties in the cocoon and B100rw hydrogels during the initial reaction. The adhesion of B100rw hydrogels increased sharply and faster than SF gel at 37 and 4°C (Fig 3c, d).

Next, we analyzed the effect of sonication time on the initial reaction of the texture profile. After sonication for the indicated times (10; 30; 60; and 1,800 s), each extract was incubated for one day at 37°C. The compressive stress in the cocoons and B100rw-B hydrogel increased slightly after sonication for 30 s and significantly in B100rw-A after 60 s. Furthermore, the compressive stress of all hydrogels increased after 1,800 s of sonication (Fig 3e). The adhesiveness of the cocoon and B100rw-C hydrogels increased after 30 s (Fig 3f). Together, these findings indicated that B100rw-A required a relatively short period of ultrasonic treatment to form a gel with increased strength.

### Proteomics analysis

**Fig 4:**
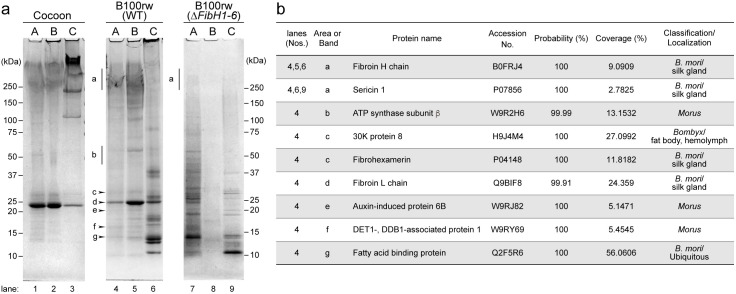
SDS-PAGE and LC-MS/MS analysis of cocoon, B100rw (WT), and B100rw (Δ*FibH1-6*). **(a)** SDS-PAGE analysis of protein components in the cocoon, B100rw (WT), and B100rw (Δ*FibH1-6*) hydrogel extracts. A, B, and C indicate methods A, B, and C are followed for sample preparation, respectively. The bands indicated by area (a, b) and arrowheads (c-g) were excised and subjected to LC-MS/MS analysis. **(b)** A list of identified proteins corresponding to peptides obtained through a database search; the probability (%) and coverage (%) in LC-MS/MS analysis are shown. LC-MS/MS, liquid chromatography-tandem mass spectrometry.

Next, we explored the protein profiles in hydrogels using SDS-PAGE (Fig 4 and S1 Raw Images). The cocoon hydrogel extract showed the presence of the SF complex consisting of FibH, Fib-L, and P25/fibrohexamerin [4]. The FibH subunit was also detected in B100rw-A, -B, and -C hydrogel extracts (Fig 4a, lanes 4, 5, 6), but not in B100rw *(*∆*FibH1-6)* extracts (Fig 4a, lanes 7, 8, 9). The sericin 1 subunit was identified as a smear in the high molecular mass region (> 250 kDa) in cocoon hydrogels [23,24] (Fig 4a, lane 3). It was also detected in B100rw (Fig 4a, lanes 4, 6) as well as B100rw (∆*FibH1-6)* hydrogels (Fig 4a, lane 9). Additionally, the identified subunits included ATP synthase subunit β [25], the 30 K protein 8 [26], auxin-induced protein 6B [27], DET1- and DDB1-associated protein1 [28], and Fatty acid binding protein [29] (Fig 4a, lane 4). These proteins were likely derived from the fat body, hemolymph, and mulberry leaves (Fig 4b). These findings indicate that B100rw hydrogels contained both larval and mulberry leaf-derived proteins, and the protein profiles varied depending on the extraction method.

### FT-IR analysis

**Fig 5:**
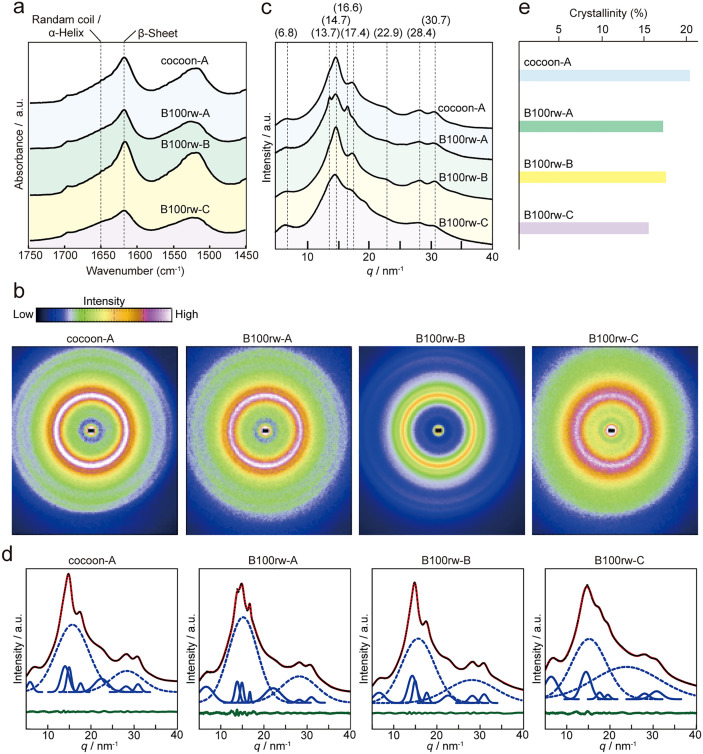
FT-IR and WAXS analysis of cocoon-A, B100rw-A, -B, and -C. **(a)** FT-IR spectra of amide I regions ranging from 1450 to 1750 cm^–1^ for the samples. **(b)** Two-dimensional WAXS profiles of the samples. **(c)** One-dimensional radially integrated WAXS profiles. **(d)** Peak separations of one-dimensional radial integration WAXS profiles using Gaussian function using Igor Pro 8.03. Black lines are fitted curves. Red dotted lines are measured curves. Broken lines represent the amorphous halos. Solid lines represent the crystal peals. Green lines show the residual between the fitted curves and measured curves. **(e)** Crystallinity of the samples.

Next, the secondary protein structures in the cocoon and B100rw hydrogels were determined using FT-IR spectroscopy. The FT-IR spectra, including the amide I region (approximately 1,680–1,580 cm^–1^) of cocoon-A, B100rw-A, -B, and -C, are shown in Fig 5a and S3 File. The peak at 1650 cm^–1^ corresponds to a random coil and helical structure. The peak at 1620 cm^–1^ indicates the formation of β-sheet crystal structures [14]. B100rw-A, -B, and -C displayed a crystalline β-sheet structure similar to cocoon-A. Additionally, the peak positions of the cocoon-A and B100rw-B were similar. However, B100rw-C exhibited a smaller proportion of β-sheets than the other hydrogels, indicating differences in the secondary structures of the hydrogels.

### WAXS analysis

WAXS analysis was performed to monitor the coherent interference of scattered X-rays from the crystal regions of the material. The crystal structure and crystallinity of cocoon-A, B100rw-A, -B, and -C were evaluated based on WAXS scattering data (Fig 5b-d and S4 File). The two-dimensional WAXS data of the gelled samples were radially integrated to produce one-dimensional scattering profiles (Fig 5b, c). In one-dimensional data, cocoon-A exhibited peaks at *q* values of 6.8, 14.7, 17.4, 22.9, 28.4, and 30.7 nm^−1^ (Fig 5c). Comparing the one-dimensional profiles of cocoon-A with those of B100rw-A, -B, and -C revealed similar peak positions, indicating that the B100rw-A, -B, and -C hydrogels are based on the SF matrix. Nevertheless, additional peaks at q values of 13.7 nm^−1^ and 16.6 nm^−1^ were observed in B100rw-A, suggesting the presence of another polymer in this hydrogel that is not found in cocoon-A. The one-dimensional WAXS profiles were further deconvoluted into amorphous and crystalline regions using Gaussian functions (Fig 5d). B100rw-A, -B, and -C exhibited lower crystallinity than cocoon-A, with B100rw-B having the closest crystallinity to that of cocoon-A (Fig 5e).

## Discussion

We successfully developed gelation protocols for B100rw (-A, -B, and -C) based on standardized methods for preparing SF hydrogel. Using the *FibH* KO mutant, we identified FibH as the primary factor responsible for gelation in all B100rw gels. SF consists of an N-terminus, a C-terminus, and a central region with a highly repetitive amino acid sequence (GAGAGS)_n_, rich in G and A. These amino acids are largely hydrophobic and tend to assemble into β-sheets in aqueous environments, stabilized by hydrogen bonds [9]. This behavior imparts gelation ability not only to SF but also to B100rw. However, the B100rw hydrogel exhibited lower compressive stress and adhesiveness than the SF hydrogel. This could be attributed to the variation in the components of LiBr extract across the protocols. Specifically, method B was more effective at removing water-soluble proteins (e.g., sericin and α-helical/random coiled silk) than method A, resulting in a gel with properties more similar to that derived from silkworm cocoons. The crystal and protein secondary structures of B100rw-B closely resembled those of cocoon-A-derived gels, consistent with its highest gel strength among the tested B100rw hydrogels. Conversely, method C retained a larger amount of water-soluble components, which likely affected the gelation process of B100rw. These components may have inhibited fibroin crystallization, as indicated by previous studies [30]. The crystallinity of B100rw-C was the lowest, which correlates with a smaller proportion of β-sheets in the FT-IR spectrum compared to the other B100rw hydrogels.

The physical gelation of SF involves the formation of β-sheet structures between adjacent chains, a process that can take a long time to stabilize under ambient conditions and is not accelerated at low temperatures [9]. In contrast, B100rw hydrogels exhibited increased compressive stress and adhesiveness, both at low tempberatures and during the rapid initial gelation phase. B100rw likely contains naturally occurring polymers such as cellulose, chitin, sericin, and fibroin, which may have influenced the properties of the hydrogels. Among these, sericin appears to affect the properties of the B100rw hydrogels significantly.

The core structure of cocoon silk fibers consists of SF layers encased by water-soluble sericin proteins. Sericins are globular, hydrophilic proteins that function as an outer, gummy coating, helping to bind the fibroin fibers together [16]. Sericin-1, −2, −3, −4, and −5 have been identified, with sericin-1 being primarily expressed during the fifth instar stage, contributing to the major sericin layers in cocoon silk. Sericin is increasingly used as a biopolymer in biomedical applications such as drug delivery, tissue engineering, and serum-free cell culture media [31]. It comprises approximately 80% hydrophilic amino acids, including serine, threonine, aspartic acid, and glutamic acid [16], and features a serine-rich repetitive sequence and serine/threonine linkage. These features play a crucial role in forming and stabilizing β-sheet aggregates by promoting intra- and intermolecular hydrogen bonding through the hydroxyl side chains [32]. Moreover, the solubility of sericin decreases with decreasing temperature, causing a transition from a random coil structure to β-sheet formation, which facilitates hydrogel formation [31].

In this study, we demonstrated that B100rw gelled faster than cocoon-A at low temperatures (4°C), suggesting that sericin, at low temperatures, acts as a gelling nucleus by promoting and stabilizing β-sheet formation. These β-sheets then form a network that facilitates the gelation of fibroin. In contrast, the lower gel strength observed in B100rw-C may be due to the higher water retention and hydrophilic components resulting from the abundance of sericin, a highly swellable protein.

Additionally, natural polysaccharides such as cellulose and chitin are known to interact with SF to promote the formation of β-sheets [33]. These polysaccharides may serve as gelling nuclei and gelation agents, facilitating the development and propagation of β- sheets, resulting in rapid gelation. Amphiphilic compounds such as surfactants and fatty acid salts are known to induce SF gelation [34–36]. Specifically, the hydrophobic region of SF interacts with amphiphiles, leading to self-assembly into micelles, which stabilizes the conformation. This electrostatic effect accelerates β-sheet formation by forcing the β-sheet motifs into closer proximity, thereby facilitating the alignment of the SF molecular structure [36]. Therefore, it is possible that the amphiphilic substances present in the larvae and mulberry leaves contributed to the increased gel strength and gelation rate of B100rw hydrogels. These findings suggest that B100rw hydrogels likely contain various gelling nuclei and gelation agents that promote the formation of β-sheets, contributing to their unique gelation behavior.

Edible insects, including silkworms, are a healthy and sustainable source of amino acids, fatty acids, and various micronutrients. Previous studies have explored the potential of gelling insect powders for further development of insect-based food [37,38]. In this study, we developed a novel hydrogel by blending silkworm-derived polymers using LiBr extraction. LiBr is highly effective in dissolving *B. mori* silk fibers and is widely used to prepare SF hydrogels [9]. However, LiBr has several limitations, including its high cost, environmental effects, and toxicity to organisms. Therefore, research is ongoing to develop alternative eco-friendly reagents for SF solubilization [39]. Additionally, several strategies have emerged to enhance SF hydrogel properties, including nanoparticle or inorganic particle reinforcement, dual physical-chemical crosslinking, blending with other polymers, double network formation, low-temperature crosslinking/cryogelation, and genetic engineering [9,40]. To effectively utilize the functional materials contained in silkworm larvae, future research should explore advanced manufacturing technologies for B100rw hydrogel construction, particularly optimized for oral administration. This could pave the way for developing functional and medicinal foods to improve the intestinal environment.

## Conclusion

In this study, we developed a novel gelation protocol for silkworm-derived hydrogels (B100rw) using various extraction methods, focusing on the effect of sericin and fibroin on gelation behavior. These findings indicate that the extraction method affects gel properties, with method B producing the strongest hydrogel due to the more effective removal of water-soluble proteins. Our study also provides evidence that sericin significantly promotes silk fibroin gelation, especially at low temperatures. Despite promising results, the limitations of this study, such as the use of toxic LiBr and restricted testing conditions, necessitate further research to optimize the hydrogel formulation and address sustainability concerns. Future studies should explore alternative extraction methods, large-scale production techniques, and biological evaluations to validate the potential applications of these hydrogels in tissue engineering, drug delivery, and sustainable food production. This research contributes to the advancement of silkworm-based hydrogels, offering valuable insights for biopolymer applications in both biomedical and food industries.

## Supporting information

S1 Fig Knockout mutant of the fibroin heavy chain (*FibH*) gene. (**a**) Schematic representations of *FibH*. Boxes and lines represent exons and introns, respectively. Light blue boxes indicate the regions of an N-terminus, C-terminus, and non-repetitive sequences. Gray boxes indicate the region with a highly repetitive amino acid sequence, (GAGAGS)_n_. The sizes of exons and introns (in bp) are indicated using scales on the map. Orange triangles represent the CRISPR target site. (**b**) Partial coding sequences corresponding to the FibH N-terminus of the wild-type (WT) and mutant (*∆FibH1-6*). The sequence of the CRISPR target site is indicated using orange boxes. The identical bases are indicated using asterisks. The *∆FibH1-6* mutant exhibits an inserted 22 bp sequence in the N-terminal region of *FibH* so that the protein encodes the 10 amino acids.
(PDF)

S1 Table Ratio of silk gland weight to body weight. After the dry weight of 10 larvae was measured, the silk glands were dissected, and their weights were calculated. The ratio of the silk gland weight was calculated to be 17.3%, accounting for approximately 1/7 of the whole body weight.
(PDF)

S1 Raw Images Original gel images of Fig 4a. (PDF)

S1 File Compressive stress (kPa) of each hydrogel prepared using the three methods in Fig 2e. (XLSX)

S2 File Compressive stress and adhesion data for hydrogels in Fig 3. (XLSX)

S3 File FT-IR analysis data in Fig 5a. (XLSX)

S4 File WAXS analysis data in Fig 5c. (XLSX)
