# Supplementary material for: Insect-derived polymer hydrogel based on fibroin matrix from whole silkworm larvae
Source: PLoS One. 2025 Nov 7;20(11):e0335864. doi: 10.1371/journal.pone.0335864 (PMC12594361; doi:10.1371/journal.pone.0335864)
Supplement: S1 Fig — (a) Schematic representations of FibH. Boxes and lines represent exons and introns, respectively. Light blue boxes indicate the regions of an N-terminus, C-terminus, and non-repetitive sequences. Gray boxes indicate the region with a highly repetitive amino acid sequence, (GAGAGS)n. The sizes of exons and introns (in bp) are indicated using scales on the map. Orange triangles represent the CRISPR target site. (b) Partial coding sequences corresponding to the FibH N-terminus of the wild-type (WT) and mutant (∆FibH1-6). The sequence of the CRISPR target site is indicated using orange boxes. The identical bases are indicated using asterisks. The ∆FibH1-6 mutant exhibits an inserted 22 bp sequence in the N-terminal region of FibH so that the protein encodes the 10 amino acids. (PDF) [file pone.0335864.s001.pdf]

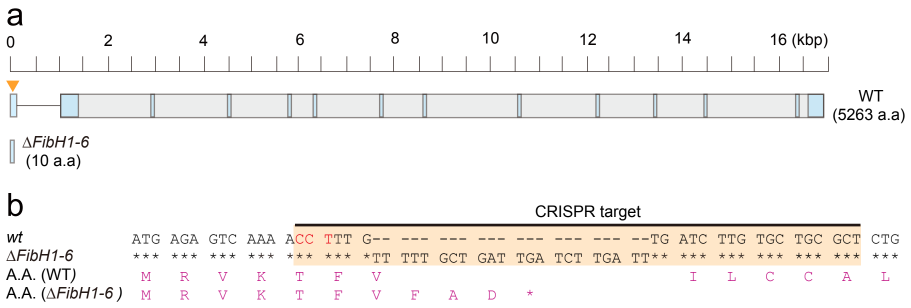

S1 Fig. Knockout mutant of the fibroin heavy chain (*FibH*) gene. (**a**) Schematic representations of *FibH*. Boxes and lines represent exons and introns, respectively. Light blue boxes indicate the regions of an N-terminus, C-terminus, and non-repetitive sequences. Gray boxes indicate the region with a highly repetitive amino acid sequence, (GAGAGS)n. The sizes of exons and introns (in bp) are indicated using scales on the map. Orange triangles represent the CRISPR target site. (**b**) Partial coding sequences corresponding to the *FibH* N-terminus of the wild-type (WT) and mutant (Δ*FibH1-6*). The sequence of the CRISPR target site is indicated using orange boxes. The identical bases are indicated using asterisks. The Δ*FibH1-6* mutant exhibits an inserted 22bp sequence in the N-terminal region of *FibH* so that the protein encodes the 10 amino acids.
