# Supplementary material for: Insect-derived polymer hydrogel based on fibroin matrix from whole silkworm larvae
Source: PLoS One. 2025 Nov 7;20(11):e0335864. doi: 10.1371/journal.pone.0335864 (PMC12594361; doi:10.1371/journal.pone.0335864)
Supplement: S1 Table — After the dry weight of 10 larvae was measured, the silk glands were dissected, and their weights were calculated. The ratio of the silk gland weight was calculated to be 17.3%, accounting for approximately 1/7 of the whole body weight. (PDF) [file pone.0335864.s002.pdf]

S1 Table. Ratio of silk gland weight to body weight.

larva	dry weight of larva (mg)	dry weight of silk gland (mg)	percent of silk gland (%)
#1	394.4	85.1	21.6
#2	332.3	54.1	16.3
#3	367.2	54.8	14.9
#4	328.3	54.9	16.7
#5	359.3	55.0	15.3
#6	388.0	81.1	20.9
#7	365.2	59.2	16.2
#8	337.7	73.2	21.7
#9	369.7	52.2	14.1
#10	354.0	55.2	15.6
Average	369.3	63.9	17.3

Ratio of silk gland weight to body weight. After the dry weight of 10 larvae was measured, the silk glands were dissected, and their weights were calculated. The ratio of the silk gland weight was calculated to be 17.3%, accounting for approximately 1/7 of the whole body weight.
