# Supplementary figures and images for: Insect-derived polymer hydrogel based on fibroin matrix from whole silkworm larvae
Source: PLoS One. 2025 Nov 7;20(11):e0335864. doi: 10.1371/journal.pone.0335864 (PMC12594361; doi:10.1371/journal.pone.0335864)

Original gel images of Fig. 4a

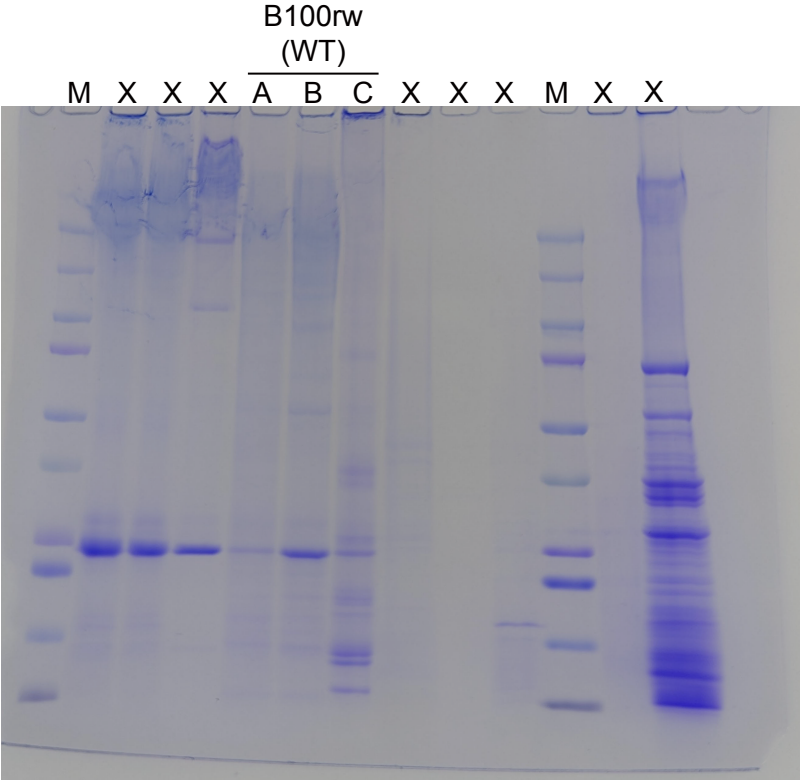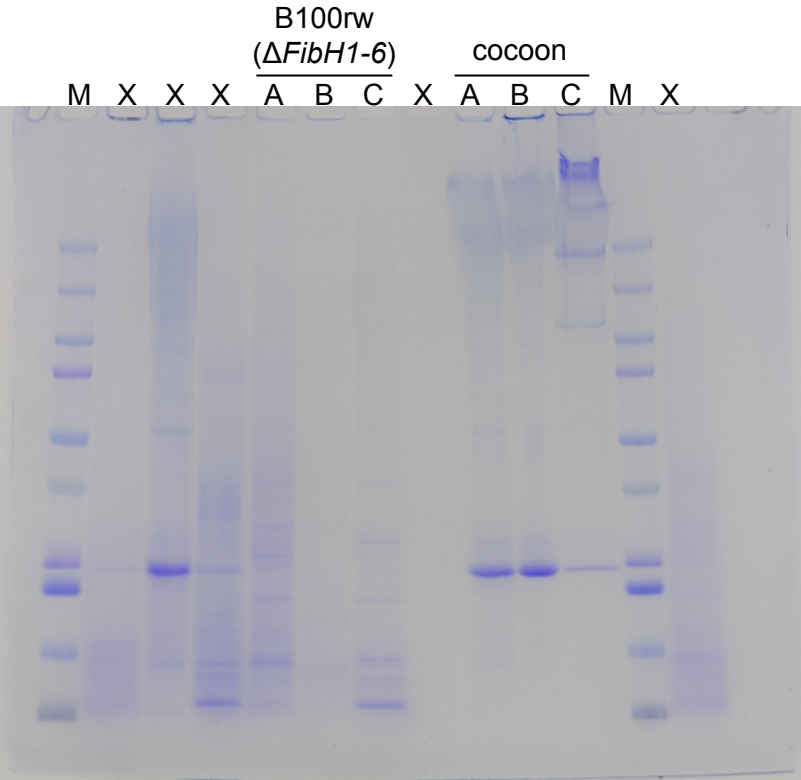

Supplement: S1 Raw Images — (PDF) [file pone.0335864.s003.pdf]
